# Oral nitrate-reducing bacteria as potential probiotics for blood pressure homeostasis

**DOI:** 10.3389/fcvm.2024.1337281

**Published:** 2024-04-04

**Authors:** Xiaofen Chai, Libing Liu, Feng Chen

**Affiliations:** ^1^ Central Laboratory, Peking University School and Hospital of Stomatology, Beijing, China; ^2^ Department of Nutrition and Health, China Agricultural University, Beijing, China

**Keywords:** hypertension, nitric oxide, oral microbiota, nitrate-reducing bacteria, probiotics, blood pressure homeostasis

## Abstract

Hypertension is a leading cause of morbidity and mortality worldwide and poses a major risk factor for cardiovascular diseases and chronic kidney disease. Research has shown that nitric oxide (NO) is a vasodilator that regulates vascular tension and the decrease of NO bioactivity is considered one of the potential pathogenesis of essential hypertension. The L-arginine-nitric oxide synthase (NOS) pathway is the main source of endogenous NO production. However, with aging or the onset of diseases, the function of the NOS system becomes impaired, leading to insufficient NO production. The nitrate–nitrite–NO pathway allows for the generation of biologically active NO independent of the NOS system, by utilizing endogenous or dietary inorganic nitrate and nitrite through a series of reduction cycles. The oral cavity serves as an important interface between the body and the environment, and dysbiosis or disruption of the oral microbiota has negative effects on blood pressure regulation. In this review, we explore the role of oral microbiota in maintaining blood pressure homeostasis, particularly the connection between nitrate-reducing bacteria and the bioavailability of NO in the bloodstream and blood pressure changes. This review aims to elucidate the potential mechanisms by which oral nitrate-reducing bacteria contribute to blood pressure homeostasis and to highlight the use of oral nitrate-reducing bacteria as probiotics for oral microbiota intervention to prevent hypertension.

## 1 Introduction

Hypertension is one of the main causes of global morbidity and mortality and poses a major risk factor for cardiovascular diseases such as heart disease, stroke, and chronic kidney disease. Recent findings of the sixth round of the national hypertension survey indicate that approximately 245 million people with hypertension in China, with an annual increase of about 10 million individuals (1). The development of hypertension is a complex process, which is influenced by genetic factors, age, dietary habits, and daily behaviors. Research has found that primary hypertension is characterized by a decrease in the production and bioavailability of nitric oxide (NO), suggesting that maintaining or restoring NO homeostasis *in vivo* may be a potential solution for the prevention and treatment of hypertension (2).

With the advancement of microbial genomics research, more studies suggest that the human microbiota, which includes symbiotic microbial communities, plays a role in various physiological processes of the host, such as energy homeostasis and cardiovascular function (3). The oral cavity, as one of the most complex microbial ecosystems in the human body, has been extensively studied for its role in oral health. There is a group of bacteria in the oral cavity that possess the ability to reduce nitrate and participate in human nitrogen metabolism through the nitrate–nitrite–NO pathway, thereby maintaining NO homeostasis and vascular dilation to alleviate hypertension in the host (4). This review provides an overview of the metabolism and effects of NO in the body and explores the role of the oral microbiota in the nitrate–nitrite–NO pathway and its potential function in maintaining host blood pressure homeostasis, aiming to provide theoretical references for exploring measures to prevent and treat hypertension.

## 2 Relationship between NO and blood pressure homeostasis

NO is an important signaling molecule in mammals, participating in various physiological processes such as endothelial function, immune function, and glucose metabolism (5). Its relationship with blood pressure homeostasis was first discovered approximately 40 years ago, and its role as a signaling molecule in the cardiovascular system for regulating vascular tone was awarded the Nobel Prize in 1998 (6). NO in the blood vessels is released by endothelial cells and significantly affects endothelial and cardiovascular physiology. The decreased bioavailability of NO is a common feature in cardiovascular diseases, and the mechanisms by which NO maintains blood pressure homeostasis are complex (7). NO exhibits lipophilic properties, allowing its diffusion from endothelial cells to vascular smooth muscle cells. It binds to the heme moiety of soluble guanylate cyclase (sGC), activating sGC to catalyze the conversion of guanosine triphosphate (GTP) to cyclic guanosine monophosphate (cGMP). The increased synthesis of cGMP leads to an increase in intracellular calcium ion flux, ultimately resulting in the relaxation of vascular smooth muscle, vasodilation, and a decrease in blood pressure (8, 9). The early discovery of nitroglycerin as an old vasodilator used in the treatment of angina pectoris demonstrated its action through the release of NO and activation of sGC, thus promoting the identification of NO function (10). Impaired endothelial function leads to imbalances in NO homeostasis, resulting in endothelium-dependent vasodilatation dysfunction. NO can protect endothelial cells from damage and inflammation by reducing the expression of adhesion molecules within these cells, thus maintaining the normal function of the vascular endothelium and preventing vascular injury and thrombus formation (11, 12). Moreover, NO inhibits platelet aggregation, reduces platelet viscosity and blood viscosity, improves hemodynamics, lowers blood pressure, and reduces the risk of cardiovascular diseases (13). NO also reduces the activity of the renin–angiotensin–aldosterone system, an important mechanism that regulates blood pressure and fluid balance (14). The biological activity of NO is closely related to kidney and metabolic functions. It can affect sodium reabsorption and potassium excretion by regulating ion channels in renal tubules, thereby influencing blood pressure stability (15). As mentioned above, NO is essential for regulating blood pressure homeostasis. However, when the immune cells perceive a threat, NO levels may increase, increasing the likelihood of cell death (16). Additionally, excessive NO generation can induce cellular oxidative stress, promoting inflammatory reactions (17). Therefore, maintaining NO homeostasis is crucial for regulating the overall physiological balance and ensuring vascular stability, particularly in the context of hypertension (18). Exploring the mechanisms and pathways involved in maintaining NO homeostasis has potential application value for the prevention and control of hypertension.

## 3 NO production pathways

### 3.1 Endogenous NO synthase pathway

**Figure 1:**
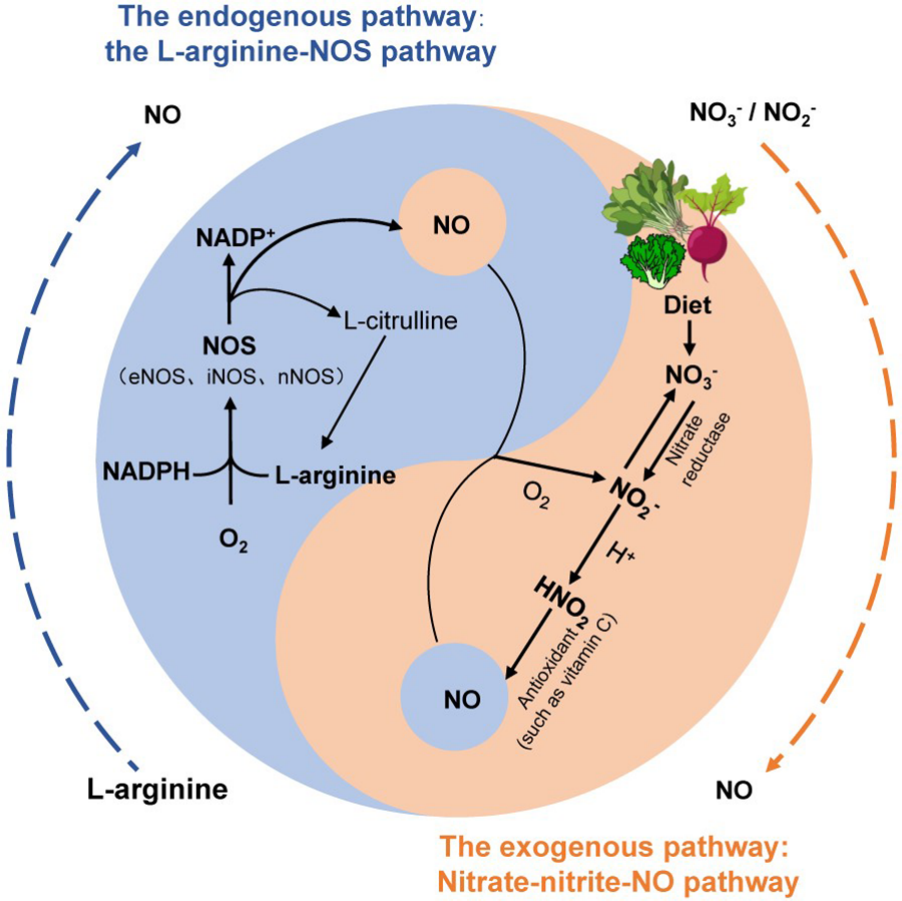
The two production pathways of NO in the human body. The endogenous pathway of NO (blue) is that L-arginine is oxidized to NO through a complex series of reactions involving nitric oxide synthase (NOS). The exogenous pathway of NO (orange) is the circulation of nitrate in humans by the nitrate–nitrite–NO pathway [modified from (5, 19)].

NO, as an important vasodilator in the human body, is responsible for arterial relaxation and dilation. Reduced generation or enhanced metabolism of NO leading to decreased bioactivity of NO is considered a potential mechanism underlying the onset of primary hypertension and is associated with aging, renal, cardiovascular, and metabolic disorders (2). Several studies have suggested that maintaining NO homeostasis in the body could be a potential solution for blood pressure control (5, 18). In endothelial cells, NO is produced by nitric oxide synthase (NOS). The classical view is that the L-arginine-NOS pathway is the major endogenous source of NO formation and signaling. Under normal physiological conditions, L-arginine and molecular oxygen generate NO through the action of NOS, maintaining the balance in the body (19). There are three isoforms of NOS, namely, endothelial NOS (eNOS), neuronal NOS (nNOS), and inducible NOS (iNOS). Among them, eNOS is mainly present in endothelial cells, where it plays a crucial role in vasodilation and improving blood flow and oxygen delivery, thus maintaining vascular health (20). With aging, there is a decrease in NOS gene expression, resulting in reduced availability of endogenous NO derived from NOS, which ultimately leads to an increased incidence of hypertension in the elderly population (21). In addition, when the body experiences local hypoxia or pathologies such as renal, cardiovascular, and metabolic diseases, NOS pathway synthesis is also hindered, and endogenous NO synthesis is impaired. In such cases, exogenous nitrate intake can effectively maintain NO homeostasis (5, 22) (Figure 1).

### 3.2 Nitrate–nitrite–NO pathway

**Figure 2:**
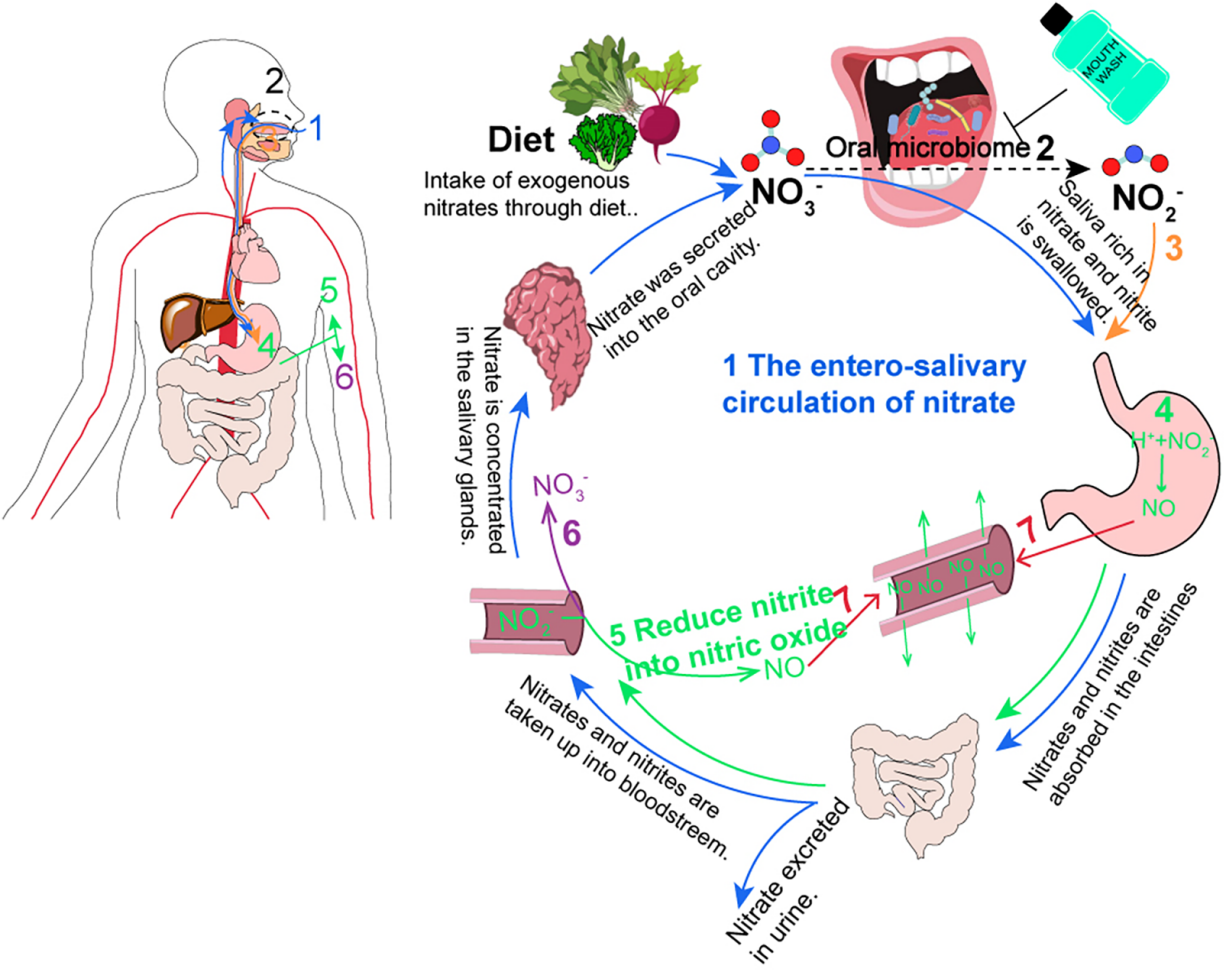
The circulation of nitrate in humans. (1) The enterosalivary circulation of nitrate (blue) [modified from (19, 29)]. (2) Oral bacteria reduce NO_3_^-^ to NO_3_^-^ (black dotted). This program becomes disrupted with antiseptic mouthwash. (3) The saliva with NO_2_^-^ is swallowed (orange). (4 and 5) In the stomach, blood, and tissues, NO_2_^-^ was reduced into NO (green). (6) NO_2_^-^ is oxidized back into NO_3_^-^ (purple). (7) NO has the benefits of vasodilation (red).

Inorganic nitrates and nitrites have been regarded as harmful food additives with potential carcinogenic effects (23). However, as research and applications progress, nitrate as a natural dietary nutrient has been extensively studied for its biological activity, particularly in disease treatment, such as in acute cardiovascular diseases (19, 24). The main exogenous source of nitrate in the human body is through diet, particularly in vegetables such as lettuce, spinach, and beetroot, which are rich in nitrate (23, 25). Therefore, the World Health Organization recommends an average daily intake of approximately 400 g of mixed vegetables (equivalent to 2.5 mmol of nitrate) per person, which can effectively lower blood pressure and improve metabolism (22). Approximately 98% of the dietary nitrate absorbed through the upper digestive tract enters the bloodstream, where exogenous and endogenous nitrate are both present (22, 24, 26). Within 60 min of nitrate supplementation, the nitrate concentration in the plasma reaches its peak and remains at a high level for a certain period of time (with a half-life of 5–6 h) (27). Wagner et al. (28) found that approximately 60% of nitrate is excreted through urine using isotopically labeled nitrate, while the remaining 25% is actively secreted into saliva by the nitrate transporter, sialin, after being enriched in the salivary glands and enters the gastrointestinal–salivary circulation system (19, 29) (Figure 2). Furthermore, the participants were fasted overnight and ingested sodium nitrate with no allowance for food or drinks during the experiment, and an increase in nitrate levels was detectable in saliva within 20–60 min of sodium nitrate (27). At this point, the nitrate level in the salivary (100–500 μM) can be 10–20 times higher than the plasma concentration (29). In the oral cavity, the high concentration of saliva nitrate is partially reduced to nitrite and NO, which are then swallowed and enter the stomach as nitrate and nitrite. In the presence of gastric acid, most of the nitrite is protonated to form HNO_2_, which further decomposes to NO and other nitrogen oxides through the action of antioxidants such as vitamin C and polyphenols. This process is known as the nitrate–nitrite–NO pathway (22, 26). Meanwhile, some nitrite enters the bloodstream and is oxidized to nitrate or is reduced to NO by various proteins and enzymes, including hemoglobin, myoglobin, xanthine oxidoreductase (XOR), and deoxyribonucleic acid (DNA) repair enzymes, in the blood and tissues (22). Therefore, during both physiological and pathological hypoxic conditions, increasing the levels of NO through the nitrate–nitrite–NO pathway ensures the production of NO even when oxygen-dependent NOS enzyme activity is impaired. This helps regulate vascular relaxation and vascular function (19), thereby increasing dietary intake of nitrate has beneficial effects on systemic cardiac metabolism.

## 4 The oral microbiota regulates blood pressure homeostasis

### 4.1 Involvement of oral microbiota in the nitrate–nitrite–NO pathway

In the natural environment, bacteria collectively drive the processes of nitrification and denitrification, completing the nitrogen cycle (30). Similarly, in humans, the nitrogen cycle involves the conversion of dietary nitrate into nitrite and NO through the nitrate–nitrite–NO pathway, with the reduction of nitrate to nitrite being a crucial step. Nitrite, with a half-life of up to 10 min in plasma and tissues, serves as a reservoir of NO in the body (24). Lundberg et al. (27) found that ingestion of a nitrate solution led to a fourfold increase in plasma nitrite levels in subjects. However, when subjects avoided swallowing after nitrate ingestion, thereby blocking the enterosalivary circulation of nitrate, the increase in plasma nitrite levels was prevented. This suggests that saliva is the primary source of nitrite in plasma. In humans and other mammals, there is a lack of specific and efficient nitrate reductase. XOR is the only known enzyme capable of reducing endogenous inorganic nitrate to nitrite under anaerobic conditions in livers (31). Therefore, oral microbial communities may play a crucial role in the process of nitrate reduction. Li et al. (32) observed an increased bacterial density toward the posterior part of the tongue, and significant nitrite production was detected only in the posterior part of the tongue, by partitioning the tongue into regions and performing Gram staining observation and bacterial isolation culture. Nitrate reduction was not detected in germ-free rats (29). Burleigh et al. (33) utilizing 16S rRNA sequencing, discovered the presence of abundant nitrate-reducing bacteria in saliva. Furthermore, bacteria in dental plaque were also found to have the capability to reduce nitrate to nitrite (34). Studies have shown that the use of antibacterial mouthwash leads to significant changes in the composition of saliva microbiota, resulting in a decrease in oral nitrite production and a significant decrease in plasma nitrite levels, leading to a decrease in the bioavailability of NO (35–37). These results suggest that there is a symbiotic relationship between the oral microbiota and the host, and their composition and activity play a decisive role in the generation of NO through the nitrate–nitrite–NO pathway, contributing to the regulation of cardiovascular and metabolic health in the human body (19, 22).

### 4.2 Role of oral microbiota in host blood pressure homeostasis

The oral cavity, as the gateway of the human body, is one of the five major microbial ecosystems of the human body, and it is related to cardiovascular diseases and metabolic health. Ko et al. (38) selected 139 oral samples and, through pyrophosphate sequencing and bioinformatic analysis of 16S rRNA, discovered a higher relative abundance of *Porphyromonas gingivalis* and *Actinobacteria* in hypertensive patients. This was associated with elevated pro-inflammatory cytokines, indicating a close correlation between oral microbiota and hypertension. Chen et al. (39) found that the diversity of oral bacteria in hypertensive patients was reduced, the relative abundance of bacteria in saliva changed, and there was a significant correlation between diastolic and systolic blood pressure. The use of antibiotics and antibacterial mouthwash products can reduce the number of oral bacteria and the content of sodium nitrite (40). Kapil et al. (41) found that the salivary nitrate reduction capacity of healthy subjects decreased after using an antibacterial mouthwash, resulting in a 90% reduction in nitrite content in the oral cavity and a 25% reduction in plasma nitrite content. This was associated with an increase in systolic and diastolic blood pressure of approximately 2–3 mmHg, which was significantly correlated with the decrease in plasma nitrite concentration (42). Thus, the imbalance of oral microbial flora can increase the risk of cardiovascular diseases such as hypertension (43). When the intake of inorganic nitrate increases, nitrate is reduced to nitrite and NO, with NO having the ability to eliminate species associated with oral diseases, such as *Porphyromonas gingivalis* (44). Additionally, the relative abundance of *Rothia* and *Neisseria* in the oral cavity significantly increases after supplementation with inorganic nitrate, and their relative abundance is correlated with the elevation of plasma NO_2_^−^ levels after nitrate supplementation (45). These findings suggest a potential link between oral microbiota and the blood pressure homeostasis of the host. The proposed mechanism is that oral bacteria reduce nitrate to nitrite, which then contributes to the maintenance of NO homeostasis in the body through the nitrate–nitrite–NO pathway in enterosalivary, thus improving hypertension (41, 45). Therefore, targeting specific oral bacteria involved in nitrate reduction to promote NO production and maintaining the homeostasis of NO in the body may be an effective strategy for the treatment of hypertension.

### 4.3 Diversity of the oral microbiome in blood pressure homeostasis

Currently, more than 770 known bacterial species have been found in the oral cavity, mainly belonging to the phyla *Actinobacteria*, *Bacteroidetes*, *Firmicutes*, *Proteobacteria*, *Spirochaetes*, and *Synergistetes* (46). The group of bacteria in the oral cavity capable of utilizing nitrate reduction to generate nitrite is collectively referred to as nitrate-reducing bacteria (47). Oral nitrate-reducing bacteria can use nitrate in saliva as an alternative electron acceptor for oxygen, effectively reducing it to nitrite through the action of nitrate reductase. This process leads to the production of NO and other closely related biologically active nitrogen oxide species, allowing for the nitrate–nitrite–NO pathway (2). The nitrate reductase Nar in oral bacteria is encoded by the genes *narX*, *narG*, *narJ*, *narH*, *narY*, *narI*, and *narW* (34, 47). Additionally, some bacteria (such as *Rothia*) also contain nitrite reductase *Nir* encoded by the genes *nirK* and *nirS*, which can further reduce nitrite to NO (4, 48, 49). Currently, abundant nitrate-reducing bacteria have been found in the oral cavity, including *Rothia*, *Neisseria*, *Actinomyces*, *Veillonella*, *Streptococcus*, *Propionibacterium*, and *Prevotella* (29, 47–49) (Table 1). Among them, *Veillonella* is the most abundant nitrate-reducing bacterial group, followed by *Actinomyces* (50, 51). The abundance of oral nitrate-reducing bacteria varies among different populations, with studies showing that *Actinomyces a*, *Rothia*, and *Neisseria* have relatively higher relative abundances in the oral cavity of healthy individuals compared to hypertensive patients, indicating that these bacteria may be beneficial for maintaining NO homeostasis and cardiovascular health-related indicators (39, 45), and the relative abundance of nitrate-reducing bacteria in the oral cavity is associated with the rate of nitrate reduction to nitrite (33). These studies indicate that when there is a sufficient number of nitrate-reducing bacteria in the oral cavity, dietary supplementation of NO_3_^-^ will continuously increase the intestinal salivary circulation, which is beneficial to the maintenance of NO homeostasis and cardiovascular health (4, 45).

**Table 1:** The predominant nitrate-reducing species of the oral microbiota in humans.

Phylum	Genus	Species
Actinobacteria	*Actinomyces*	*A. georgiae*, *A. graevenitzii*, *A. hongkongensis*, *A. johnsonii*, *A. lingnae*, *A. massiliensis*, *A. naeslundii*, *A. oris*, *A. viscosus*
Actinobacteria	*Rothia*	*R. aeria*, *R. dentocariosa*, *R. mucilaginosa*
Proteobacteria	*Neisseria*	*N. elongate*, *N. flavescens*, *N. macacae*, *N. mucosa*, *N. oralis*, *N. sicca*, *N. subflava*
Bacteroidetes	*Prevotella*	*P. melaninogenica*, *P. salivae*
Firmicutes	*Veillonella*	*V. dispar*, *V. parvula*, *V. atypica*, *V. tobetsuensis*
Firmicutes	*Streptococcus*	*S. infantis*, *S. salivarius*, *S. sanguinis*, *S. oralis*, *S. parasanguinis*, *S. mitis*

People are concerned about the safety of inorganic nitrate and the application of oral nitrate-reducing bacteria, primarily due to the worry that nitrite, derived from nitrate metabolism, may lead to the formation of nitrosamines, potentially leading to cancer (52). The production of nitrite depends on reaction mechanisms, compound concentrations, pH values of the reaction environment, and other regulatory factors (53). Additionally, endogenous nitrosation can be inhibited, for example, by reducing nitrite to NO through dietary compounds such as ascorbic acid, polyphenols, vitamin C, and vitamin E, thereby suppressing the formation of nitrosamines (54). Although the concentration of nitrite derived from therapeutically used nitrates and oral microbiota reduction is far below the risk level, and the production of nitrosamines is negligible, potential nitrosamine formation and damage after nitrate-reducing probiotic applications should be monitored in practice (55). Therefore, the safe and effective approach of using nitrate-reducing probiotics in combination with a balanced diet supplementing nitrate is emphasized (54). It is crucial to note that some bacteria with nitrate-reducing capabilities also possess pathogenic properties, such as *Staphylococcus aureus* (56). Therefore, when applying oral nitrate-reducing bacteria in the future, the safety of bacterial strains must be carefully considered.

## 5 The future of oral probiotics in hypertension

NO, as a vasodilator, maintains blood pressure homeostasis by controlling and regulating vascular tone. The disruption of NO homeostasis may contribute to the development of hypertension. However, supplementation with exogenous nitrate can help maintain NO homeostasis through the nitrate–nitrite–NO pathway in the enterosalivary circulation. This pathway relies on oral nitrate-reducing bacteria that convert nitrate to nitrite or NO. The net ability of subgingival plaque bacteria to generate nitrite is associated with a lower risk of cardiovascular metabolic outcomes (48). The oral microbiome shows substantial potential in disease prevention, diagnosis, and treatment (57). Therefore, in the future disease prevention and treatment, targeted application of oral nitrate-reducing bacterial communities, combined with the consumption of foods and vegetables rich in nitrates. This alternative pathway is a practical and effective strategy to promote NO production and maintain NO homeostasis in the body, which in turn may alleviate disease symptoms and reduce the incidence and severity of conditions, providing a novel approach to disease prevention and treatment.
